# Sociodemographics and Digital Health Literacy in Using Wearables for Health Promotion and Disease Prevention: Cross-Sectional Nationwide Survey in Germany

**DOI:** 10.1007/s10935-024-00821-y

**Published:** 2024-12-18

**Authors:** Chen-Chia Pan, Karina Karolina De Santis, Saskia Muellmann, Stephanie Hoffmann, Jacob Spallek, Nuria Pedros Barnils, Wolfgang Ahrens, Hajo Zeeb, Benjamin Schüz

**Affiliations:** 1 Leibniz Institute for Prevention Research and Epidemiology – BIPS, Achterstraße 30, 28359 Bremen, Germany; 2 Leibniz ScienceCampus Digital Public Health, Bremen, Germany; 3 Institute for Public Health and Nursing Research, University of Bremen, Bremen, Germany; 4 Department of Public Health, Brandenburg University of Technology (BTU) Cottbus-Senftenberg, Senftenberg, Germany; 5 Lusatian Center for Digital Public Health, Brandenburg University of Technology (BTU) Cottbus-Senftenberg, Senftenberg, Germany

**Keywords:** Digital Health, Digital Divide, Fitness Trackers, Health Technology, Consumer Behavior, Health Literacy

## Abstract

**Background:**

Wearable technologies have the potential to support health promotion and disease prevention. However, it remains unclear how the role of social determinants of health (SDoH) and digital determinants of health (DDoH) plays in this context.

**Objective:**

This study investigates differences in sociodemographic factors and digital health literacy between wearable users and non-users, whether the association with wearable use varies across age groups and its potential mediator.

**Methods:**

A cross-sectional nationwide telephone survey was conducted in November 2022 in a panel of adult internet users in Germany. Assessments included self-reported wearable use, sociodemographic factors (sex, age, education, household size and income, and residence region), and digital health literacy (measured with the eHealth Literacy Scale, eHEALS). Associations between wearable use, sociodemographic factors and digital health literacy were analyzed using binomial logistic regression models in the total sample and with age group stratification, with a supplementary mediation analysis examining digital health literacy as a mediator in the relationship between age and wearable use.

**Results:**

Overall, 24% (223/932) of participants (52% male, mean age 55.6 years) reported using wearables for health. Wearable use was lower among participants aged 65 and above, with lower educational attainment, living in 1–2 person households, with below-average household income, and residing in smaller cities or former East Germany. Wearable use prevalence is substantially lower in older age groups (*18–40*: 36%; *41–64*: 26%; *65+*:14%). Wearable users reported higher levels of digital health literacy (mean: 30.7, SD = 5) than non-users (mean: 28.3, SD = 6). Stratified analyses indicate that the association between digital health literacy and wearable use varies by age group, with significant positive association observed in older age groups (OR = 1.00, 95% CI: 0.94 to 1.07 in age group 18–40; OR = 1.07, 95% CI: 1.03 to 1.12 in age group 41–64; OR = 1.11, 95% CI: 1.04 to 1.19 in age group 65+). Mediation analysis indicated that digital health literacy partially mediates the relationship between age and wearable use (indirect effect: coefficient = -0.0156, 95% CI: -0.0244 to -0.00791, *p* <.001).

**Conclusions:**

This study indicates sociodemographic disparities in wearable use among the German population and differences in digital health literacy between wearable users and non-users. A generational divide in wearable use was identified, with older adults being less likely to embrace this technology. This was especially true for older adults with lower digital health literacy. Future public health initiatives employing health technologies should take SDoH and DDoH into consideration to ensure effective and equitable impacts.

## Introduction

### Wearable Technologies for Health Promotion and Disease Prevention

Wearable technologies with built-in multisensors that can detect skin temperature, measure blood flow, and track movement have great potential to promote individual health (Peake et al., 2018). Wearables may offer personalized health monitoring and behavior tracking (Vetrovsky et al., 2022), deliver immediate feedback on various health metrics, encourage healthier lifestyles, and support physical fitness management (Brickwood et al., 2019; Wang et al., 2020). Wearables may also have the potential to support disease prevention through screening and early detection, assist chronic disease management through remote patient monitoring and personalized care (Huhn et al., 2022), and facilitate health promotion with data-driven tailored behavior change interventions for physical activity and weight management (Wang et al., 2020).

Approximately 1.1 billion wearable devices were in use in 2022 globally, and in Germany, about 1 in 4 internet users used wearables in 2022 (Eurostat, 2023). However, their integration into healthcare systems comes with challenges, particularly regarding social differences in access and usage – the so-called “digital divide,” where disparities in access to and use of technologies may exacerbate existing health inequalities (Scheerder et al., 2017). Individuals without access to or familiarity with wearable technologies may be excluded from effective prevention and health promotion tools, widening health disparities between demographically and socioeconomically advantaged and disadvantaged populations (Lee et al., 2021). This raises questions of health equity and fairness, as those who can afford and access wearables may benefit disproportionately compared to marginalized communities (Canali et al., 2022). Furthermore, adopting wearables for health monitoring may inadvertently exacerbate disparities if certain populations are excluded from participation in research studies or clinical trials due to a lack of access or technological competencies (Kim et al., 2023). Additionally, chronic diseases disproportionately affect individuals with lower socioeconomic status as highlighted by the German Health Update (GEDA) study (Allen et al., 2021). These disparities underline the importance of understanding how sociodemographic factors influence the adoption of health-promoting technologies like wearables, which may help address health inequities.

### Sociodemographic Factors and Digital Health Literacy

Based on the Digital Health Equity Framework (Richardson et al., 2022), digital determinants of health (DDoHs) and social determinants of health (SDoHs) are intertwined factors that influence an individual’s ability and opportunity to engage with digital health tools. This framework emphasizes that individual-level factors, such as sociodemographics and digital health literacy, should be considered in product development and intervention design by digital health stakeholders with equity and impact in mind (Richardson et al., 2022). Applying this framework, our study examines how these individual-level determinants impact wearable use, identifying disparities in adoption that may contribute to health inequities across sociodemographic groups.

Previous studies show substantial sociodemographic differences in wearables uptake and usage, particularly regarding differences in age, education, income, and residence location (urban versus rural). For instance, a study from the HealthStyles 2015 survey in the US found that wearable use decreased with advancing age, lower levels of education, and in rural areas (Omura et al., 2017). Additionally, studies using the US Health Information National Trends Survey identified older age, lower education, and lower income to be associated with low wearable use across diverse populations, such as adults with diabetes, those at risk for cardiovascular diseases, and the general adult population (Dhingra et al., 2023; Kekere et al., 2020; Ranganathan et al., 2020). Moreover, a recent cohort study in the US indicated that children from lower socioeconomic status households were less likely to participate in a wearable study and wore wearables for significantly shorter durations than those from higher socioeconomic status households (Kim et al., 2023). The findings from these studies underscore the disparities in wearable use among socioeconomically and demographically disadvantaged groups, emphasizing the pressing need to prevent further widening of the digital divide (Scheerder et al., 2017).

At the same time, individual resources for the use of digital tools, such as digital health literacy, are unequally distributed along socioeconomic factors (Estrela et al., 2023), which may further exacerbate social differences in relation to health and healthcare. Digital health literacy, defined as the ability to seek, understand, evaluate, and apply health information from electronic sources to make informed decisions about health (van Kessel et al., 2022), is essential for individuals to engage with digital health services, navigate complex health data, and effectively adopt technologies for health management (Knitza et al., 2020). It has been related to better health outcomes, treatment adherence, motivation, adaptive health behaviors, and higher trust in healthcare providers (van Kessel et al., 2022). However, a recent systematic review has highlighted individual and social differences in digital health literacy: Higher education, income, and social support are associated with higher digital health literacy, whereas older age is associated with lower levels of digital health literacy (Estrela et al., 2023).

Only a small number of studies on wearable use have taken both sociodemographic factors and digital health literacy into consideration. For instance, studies from China and South Korea suggest that lower digital health literacy is often observed in older adults, individuals with lower education levels, and those residing in rural areas (Jang & Je, 2022; Liu et al., 2022). A study among US older adults (over 65) indicates that wearables use is positively associated with digital fluency, health status, education, and income (Ranganathan et al., 2021).

In Germany, a substantial portion of the population reports low digital health literacy, mostly individuals with low education, older age, and low socioeconomic status (Schaeffer et al., 2023). Additionally, unlike other European countries, Germany’s digitalization level is below the EU average, and Germany has one of the highest proportions of older adults in Europe, who may face specific barriers to digital health literacy and technology adoption (Statistisches Bundesamt (Destatis), 2023). However, no previous study has investigated how digital health literacy and sociodemographic factors together impact wearable use for health purposes in Germany, making this study an important contribution to filling the evidence gap to understand digital health equity in the German population.

### Research Aims

This study aims to investigate the role of sociodemographic factors and digital health literacy in the adoption of wearable technologies for health promotion and disease prevention among adults in Germany. Specifically, the study examines: (1) differences in sociodemographic factors and digital health literacy between wearable technology users and non-users; (2) whether the association with wearable use varies across age groups; and (3) whether digital health literacy mediates the association between age and wearable use. The study hypothesizes that:

1. Wearable users have higher digital health literacy and are more likely to belong to certain sociodemographic groups (e.g., younger age, higher education, higher income) than non-users.
2. The association between digital health literacy and wearables is positive and consistent across age groups.
3. Digital health literacy mediates the association between age and wearable use.

## Methods

### Study Design

The study is based on data from a nationwide cross-sectional survey in Germany conducted in 2022 (De Santis et al., 2024). A protocol for this study was prospectively registered(De Santis et al., 2022). Here, we provide a brief overview of the recruitment settings, participants, and measures, with a more comprehensive description of the methods available in a project report (De Santis et al., 2024). Notably, the analysis presented in this study focuses on wearable use data, which has not been previously reported.

### Recruitment Setting & Participants

The participants were recruited from a nationwide sample through an existing panel managed by Cerner Enviza GmbH (Munich, Germany). The recruitment strategy employed a random digit dialing approach, reaching both landline and mobile-only users based on their geographical region. This approach sought to achieve approximate representative population density across geographical regions in the total sample, without applying complex survey sampling elements, such as strata, primary sampling units, structure nesting, or sampling weights. While this strategy aimed to capture diverse sociodemographic representation, it does not ensure full representativeness of the general population, particularly among individuals without internet access or those with limited exposure to digital technologies. The eligibility criteria for study participants were: (1) aged 18 years or older, (2) internet user, (3) residing in Germany, and (4) able to communicate in German. The data were collected from 1,020 eligible participants in November 2022 by Cerner Enviza GmbH, using computer-assisted telephone interviews with experienced interviewers. The interview process was stopped upon reaching the desired sample size. As a result, response rate data were not recorded or made available. All interviews were conducted in German and lasted approximately 15 min (De Santis et al., 2024).

### Measures

The interview was based on a self-developed questionnaire with 30 items to assess self-reported technology use for health purposes, digital health literacy, and sociodemographic characteristics. This study focuses specifically on the adoption of wearable technology. Two items in the questionnaire involved wearable use: “I use the internet via… to stay healthy or to improve my health” and “What types of digital technologies do you use to be physically active?”. The multi-selectable answer options were (a) computer or laptop, (b) smartphone or tablet, (c) activity tracker or smartwatch, (d) gaming console, or (e) “don’t know/no answer.” Participants who reported using activity trackers or smartwatches in response to either of these items were classified as wearable users. Participants who did not report activity tracker or smartwatch use were classified as non-users. Participants were dichotomized into wearable users and non-users to align with the study’s primary objective of understanding the factors associated with wearable adoption as a distinct technological innovation.

*Digital health literacy* was measured using the eHealth Literacy Scale (eHEALS) (Norman et al., 2006). The eHEALS is a validated instrument with high internal consistency (Cronbach’s alpha = 0.88) and established construct validity in various populations, including German-speaking samples (Soellner et al., 2014). This scale reliably assesses individuals’ perceived ability to seek, understand, and evaluate digital health information. The eight items from eHEALS were self-rated by the participant on a 5-point Likert scale (from 1 = strongly disagree to 5 = strongly agree). We computed the eHEALS sum score as a continuous variable of the digital health literacy of each participant, which can range from 8 points (lowest) to 40 points (highest).

*Sociodemographic data* included age (18–40, 41–64, 65+ years), sex (female, male), education (low, medium, high) (Federal Ministry of Education and Research - BMBF, 2016), household size (1–2 persons, 3+ persons) and household income (lower than average in Germany, average or above) (Statistisches Bundesamt (Destatis), 2023), and residence region by city size (small city, medium city, large city) (Bundesinstitut für Bau- Stadt- und Raumforschung, 2021) and federal state (former East Germany, former West Germany). These variables were measured using standardized survey methods and categorized based on established frameworks for sociodemographic analysis (De Santis et al., 2024).

These covariates were selected based on their theoretical relevance within the Digital Health Equity Framework (Richardson et al., 2022) as DDoHs (digital health literacy) and SDoHs (Sociodemographic) both are critical for understanding disparities in digital health literacy and technology adoption.

### Statistical Analyses

All analyses were conducted via Jamovi version 2.3, an open-source software built on the R statistical language (R Core Team, 2021; The jamovi project, 2022). Participants with incomplete data on eHEALS items (88/1020) were excluded, resulting in a final sample size of *n* = 932 for all analyses. In the first part of the analyses, descriptive data were reported as frequencies and percentages to summarize the characteristics of the study sample and the prevalence of wearable use. Participants were categorized by wearable use (users vs. non-users) and descriptively compared using means and standard deviations (SD) for age and digital health literacy. The second part of the analyses is a total sample binomial logistic regression. This analysis included digital health literacy and all sociodemographic factors (i.e., age, sex, education, household size, household income, city size, and region/state) as mutually adjusted confounders to account for potential confounding effects. The purpose was to examine their associations with wearable use and control for confounding bias. The results are reported as adjusted odds ratios (OR) and 95% confidence intervals (CI). To assess the potential multicollinearity of sociodemographic factors and digital health literacy, the variance inflation factor (VIF) and tolerance of all predictors were calculated, and results (VIF < 2 and tolerance > 0.9) indicated a lack of multicollinearity. The third part of the analyses is subgroup binomial logistic regressions to assess the association between wearable use with digital health literacy stratified by age group. To assess potential selection bias, we compared participants excluded due to incomplete eHEALS data with those included in the final sample. Comparisons were made using chi-square tests for categorical variables and t-tests for continuous variables, examining key sociodemographics such as age, sex, education, and income. Additionally, a mediation analysis was performed to explore whether digital health literacy mediates the relationship between age and wearable use, both the direct effect of age on wearable use and the indirect effect mediated by digital health literacy were reported as coefficients and 95% CIs.

## Results

The study sample included 932 adult internet users who completed all survey items. The average age was 55.6 years (SD = 16.1), with 19% of participants born in the “Digital Native” generation (18 to 40 years of age) (Prensky, 2001). The opposite of “Digital Native” is the “Digital Immigrants”, which was made up of *Gen X* (41 to 64 years of age; 49%) and the *Silver Generation* (65+ years of age; 32%) (Wójcik-Czerniawska, 2024). Over half of the participants (52%) were male, and around 45% of the participants reported household net income equal to or above the country average (i.e., 3,500 EURO/month). Most participants completed secondary education (78%), living in a 1–2 persons household (70%), and residing in urban areas (more than 50,000 inhabitants; 80%) and in federal states of former West Germany (81%). The average digital health literacy score was 28.9 (SD = 6.2, range = 8–40).

Participants excluded due to incomplete eHEALS data (*n* = 88) were significantly older (mean age = 65.2 years, SD = 16.4) compared to those in the final sample (mean age = 55.6 years, SD = 16.1; *p* <.001). No significant differences were observed in gender, education, or income between these groups (*p* >.05 for all comparisons). This difference may introduce selection bias, which is addressed in the Discussion.

### Disparities in Wearable Use & Digital Health Literacy

Among 932 participants, less than a quarter (24%, 223/932) report using wearables for health, such as activity trackers and smartwatches. Table 1 presents the prevalence of wearable use across various sociodemographic categories alongside their corresponding digital health literacy levels. Notably, the prevalence of wearable use is lower among participants aged 65 and above, with low education, living in 1–2 person households, with below-average household income, and residing in smaller cities or former East Germany, compared to the overall prevalence. This evidence suggests sociodemographic disparities in wearable use among the German population. Additionally, the substantially lower wearable use prevalence in the older age groups is noteworthy. Hence, wearable users skewed towards a younger age profile, with an average age of 50.3 years (SD = 15.6), compared to non-users whose average age is 57.3 years (SD = 15.9). In terms of digital health literacy, there are noticeable variations in the digital health literacy scores across different sociodemographic groups in the total study sample. Participants aged 65 and above, with low education, and residing in smaller cities show lower levels of digital health literacy. Furthermore, wearable users exhibit higher levels of digital health literacy than non-users across all sociodemographic groups (see Table 1).

**Table 1:** Digital health literacy and prevalence of wearable use for health promotion and disease prevention among German adult internet users (*n* = 932, nationwide telephone interview survey) by sociodemographics

	Total sample		Wearable users		Non-users
	*n*	DHL mean (SD)	Prevalence	DHL mean (SD)	DHL mean (SD)
**Total**	932	28.9 (6.2)	23.9%	30.7 (5.3)	28.3 (6.5)
**Sex**					
Male	483	28.4 (6.3)	23.8%	30.4 (5.3)	27.8 (6.5)
Female	449	29.4 (6.0)	24.1%	31.0 (5.2)	28.8 (6.1)
**Age**					
18–40	177	30.9 (5.1)	35.6%	31.0 (4.6)	30.9 (5.4)
41–64	458	28.9 (5.9)	25.8%	30.7 (5.9)	28.4 (5.8)
65+	297	27.6 (6.9)	14.1%	30.5 (4.5)	27.1 (7.1)
**Education** ^a^					
Low	112	25.9 (6.9)	16.1%	27.7 (5.6)	25.5 (7.1)
Medium	472	28.8 (6.2)	25.0%	31.0 (5.4)	28.1 (6.3)
High	348	29.9 (5.5)	25.0%	31.0 (4.9)	29.5 (5.7)
**Household size**					
1–2 Persons	656	28.7 (6.3)	21.2%	30.7 (5.2)	28.1 (6.4)
3 Persons or more	276	29.3 (5.9)	30.4%	30.8 (5.4)	28.7 (6.0)
**Household income** ^b^					
Lower than average	393	28.5 (6.3)	22.6%	30.7 (5.2)	27.8 (6.4)
Average or above	425	29.4 (5.8)	27.1%	30.8 (5.1)	28.9 (6.0)
Prefer not to answer	114	28.4 (7.0)	16.7%	30.4 (6.6)	28.0 (7.1)
**City size** ^c^					
Small city	189	27.7 (7.1)	15.9%	30.9 (5.5)	27.1 (7.2)
Medium city	341	29.1 (5.9)	27.0%	29.7 (5.5)	28.8 (6.0)
Large city	402	29.3 (5.9)	25.1%	31.6 (4.8)	28.5 (6.0)
**Region state**					
Former East Germany	176	28.6 (6.3)	20.5%	29.5 (5.9)	28.3 (6.4)
Former West Germany	756	29.0 (6.1)	24.7%	31.0 (5.1)	28.3 (6.3)

DHL, digital health literacy; SD, standard deviation

a. ISCED 2011 – International Standard Classification of Education, Federal Ministry of Education and Research (BMBF). Low = ISCED 0–2; Medium = ISCED 3–4; High = ISCED 5–8

b. The average net household income in Germany was about 3,500 EURO/month in 2021, according to the Federal Statistical Office of Germany

c. Types of cities and municipalities in Germany, Federal Office for Building and Regional Planning (BBSR). Small city < 50,000 inhabitants; Medium city 50,000 to 500,000 inhabitants; Large city > 500,000 inhabitants

### Predictors of Wearable Use

As shown in Table 2, the total sample binomial logistic regression analysis found that age, city size, and digital health literacy are significantly associated with wearable use for health among adult internet users in Germany. Specifically, the findings indicate that older adults, particularly those aged 65+ are 60% less likely to use wearables (OR = 0.41, 95% CI: 0.26 to 0.67) compared to the younger adults in the “Digital Natives” generation (aged 18 to 40), even after accounting for other sociodemographic factors and digital health literacy (Wójcik-Czerniawska, 2024). This suggests a generational divide in wearable use, with older adults less likely to embrace this technology. Additionally, residing in medium-sized cities (50,000 to 500,000 inhabitants) is associated with an 80% higher likelihood of wearable use (OR = 1.80, 95% CI: 1.12 to 2.89) compared to residing in small cities (< 50,000 inhabitants) when controlling for other predictors. This suggests the possibility of disparities in technology adoption and access to health technologies between urban and rural areas. Moreover, higher digital health literacy significantly correlates with an increased likelihood of wearable use (OR = 1.06, 95%CI: 1.03 to 1.10). Conversely, factors such as sex, education, household size and income, or region state showed no statistical significance in their association with wearable use among adults in Germany.

**Table 2:** Adjusted odds ratios (95% confidence intervals) of using wearables for health promotion and disease prevention by sociodemographic factors and digital health literacy from the binomial logistic regression for the total sample of adult internet users in Germany (*n* = 932, nationwide telephone interview survey)

Predictor	Wearable use (ref: non-user)		
	Adjusted OR	95% CI	
		Lower	Upper
**Sex**			
Male	ref	-	-
Female	0.97	0.70	1.34
**Age**			
18–40	ref	-	-
41–64	0.74	0.51	1.10
65+	**0.41**	**0.26**	**0.67**
**Education** ^a^			
Low	ref	-	-
Medium	1.38	0.78	2.45
High	1.32	0.73	2.39
**Household size**			
1–2 Persons	ref	-	-
3 Persons or more	1.31	0.91	1.88
**Household income** ^b^			
Lower than average	ref	-	-
Average or above	1.01	0.71	1.44
Prefer not to answer	0.63	0.36	1.13
**City size** ^c^			
Small city	ref	-	-
Medium city	**1.80**	**1.12**	**2.89**
Large city	1.56	0.98	2.49
**Region state**			
Former East Germany	ref	-	-
Former West Germany	1.20	0.79	1.82
**Digital health literacy** (cont)	**1.06**	**1.03**	**1.10**

OR, odds ratio; CI, confidence interval Cont., continuous variable

The model is based on binomial logistic regression; all predictors are mutually adjusted

Bold numbers indicate statistical significance based on 95% CIs

a. ISCED 2011 – International Standard Classification of Education, Federal Ministry of Education and Research (BMBF). Low = ISCED 0–2; Medium = ISCED 3–4; High = ISCED 5–8

b. The average net household income in Germany was about 3,500 EURO/month in 2021, according to the Federal Statistical Office of Germany

c. Types of cities and municipalities in Germany, Federal Office for Building and Regional Planning (BBSR). Small city < 50,000 inhabitants; Medium city 50,000 to 500,000 inhabitants; Large city > 500,000 inhabitants

**Fig. 1:**
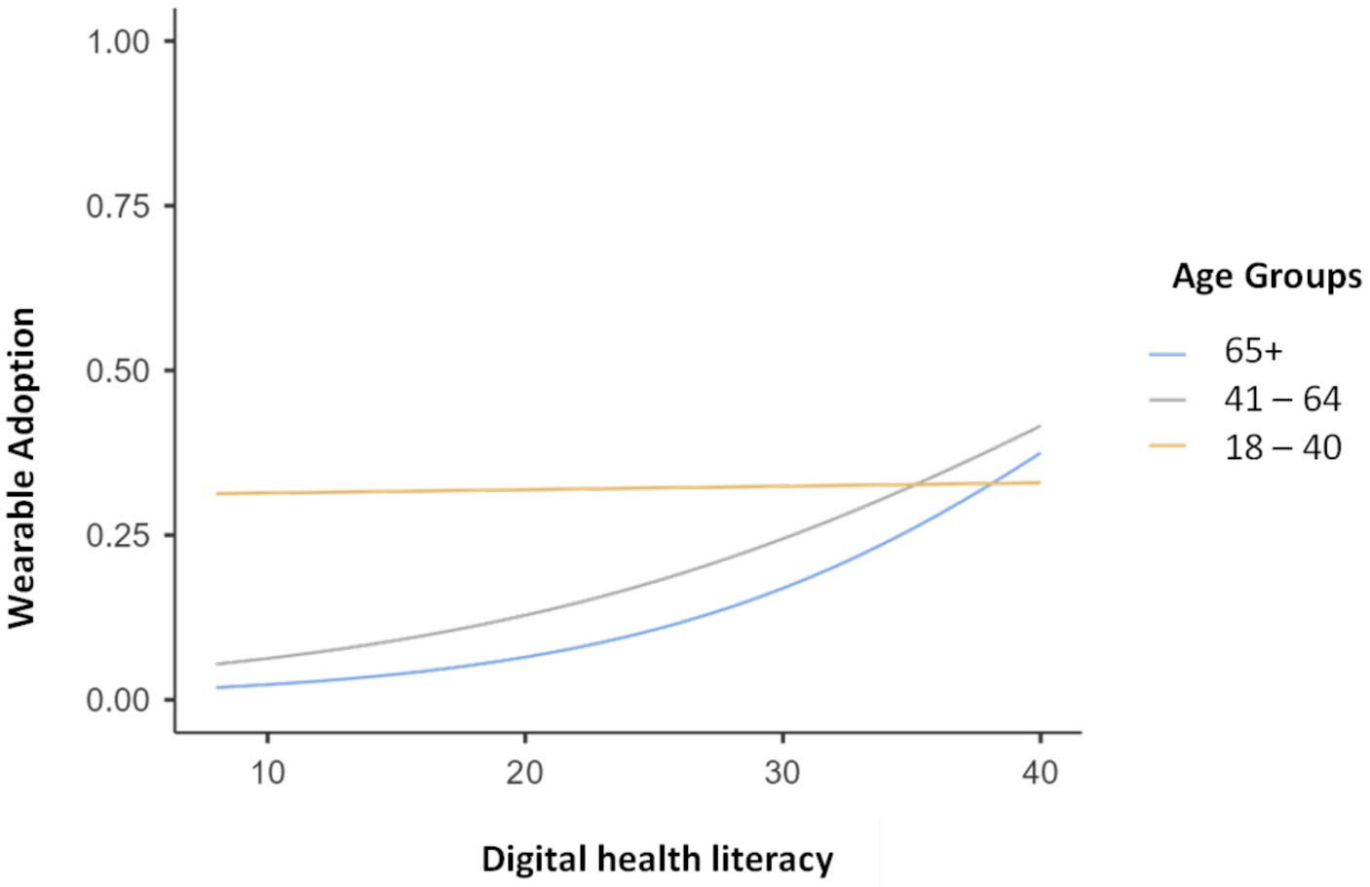
Predicted probability plots of wearable use (= 1) by digital health literacy at different age groups

To further examine the generational divide, we employed subgroup binomial logistic regressions stratified by age group (18 to 40; 41 to 64; 65+) to assess the role of sociodemographic factors and digital health literacy in wearable use (see Table 3). The results of the subgroup analysis show that digital health literacy plays different roles in wearable use across age groups. Among the “Digital Native” generation (aged 18 to 40) (Prensky, 2001), the highest prevalence of wearable use, digital health literacy is not correlated with wearable use (OR = 1.00, 95% CI: 0.94 to 1.07). In contrast, digital health literacy is a significant positive predictor of wearable use among “Digital Immigrants”, both *Gen X* (aged 41 to 64, OR = 1.07, 95% CI: 1.03 to 1.12) and the *Silver Generation* (aged 65+, OR = 1.11, 95% CI: 1.04 to 1.19) (Wójcik-Czerniawska, 2024). Figure 1 shows the predicted probability of wearable use across levels of digital health literacy for each age group. The plots reveal that wearable use is high among Digital Natives regardless of their digital health literacy (Prensky, 2001). These visualizations provide a clearer depiction of the association between digital health literacy and wearable use across different age demographics. This evidence suggests that older adults with higher digital health literacy are substantially more likely to use wearable for health promotion and disease prevention than those with lower digital health literacy. Additionally, for adults aged 41–64, living in a large city increases the likelihood of using wearables (OR = 1.88, 95% CI: 1.01 to 3.50). This suggests that urban-rural differences do not significantly affect the high prevalence of wearable use in younger adults or the low prevalence in older adults.

**Table 3:** Adjusted odds ratios (95% confidence intervals) from the subgroup binomial logistic regressions assessing associations between wearable use for health promotion and disease prevention with sociodemographic factors and digital health literacy by age groups

Predictor	Wearable use (ref: non-user)		
	18 to 40, *n* = 177	41 to 64, *n* = 458	65+, *n* = 297
	Adjusted OR (95% CI)	Adjusted OR (95% CI)	Adjusted OR (95% CI)
**Sex**			
Male	ref	ref	ref
Female	1.61 (0.82–3.16)	0.89 (0.57–1.39)	0.87 (0.43–1.76)
**Education** ^a^			
Low	ref	ref	ref
Medium	2.21 (0.56–8.76)	1.95 (0.85–4.45)	0.51 (0.18–1.46)
High	2.08 (0.51–8.53)	1.46 (0.61–3.51)	0.72 (0.25–2.09)
**Household size**			
1–2 Persons	ref	ref	ref
3 Persons or more	1.02 (0.49–2.11)	1.34 (0.85–2.11)	0.97 (0.19–4.84)
**Household income** ^b^			
Lower than average	ref	ref	Ref
Average or above	0.86 (0.42–1.77)	1.30 (0.77–2.18)	0.79 (0.38–1.67)
Prefer not to answer	0.65 (0.17–2.56)	1.02 (0.49–2.14)	0.13 (0.02–1.00)
**City size** ^c^			
Small city	ref	ref	ref
Medium city	1.47 (0.49–4.38)	1.63 (0.87–3.05)	1.95 (0.71–5.37)
Large city	1.64 (0.57–4.74)	**1.88 (1.01–3.50)**	1.43 (0.51–4.00)
**Region state**			
Former East Germany	ref	ref	ref
Former West Germany	2.16 (0.88–5.30)	1.01 (0.56–1.85)	0.88 (0.38–2.04)
**Digital health literacy** (cont)	1.00 (0.94–1.07)	**1.07 (1.03–1.12)**	**1.11 (1.04–1.19)**

OR, odds ratio; CI, confidence interval; Cont, continuous variable

All predictors are mutually adjusted. Bold numbers indicate statistical significance based on 95% CIs

a. ISCED 2011 – International Standard Classification of Education, Federal Ministry of Education and Research (BMBF). Low = ISCED 0–2; Medium = ISCED 3–4; High = ISCED 5–8

b. The average net household income in Germany was about 3,500 EURO/month in 2021, according to the Federal Statistical Office of Germany

c. Types of cities and municipalities in Germany, Federal Office for Building and Regional Planning (BBSR). Small city < 50,000 inhabitants; Medium city 50,000 to 500,000 inhabitants; Large city > 500,000 inhabitants

A mediation analysis was conducted to explore whether digital health literacy mediates the relationship between age and wearable use. The indirect effect of age on wearable use through digital health literacy was significant (coefficient = -0.0156, 95% CI: -0.0244 to -0.00791, *p* <.001), suggesting that older age is associated with lower digital health literacy, which in turn is associated with lower wearable use. The direct effect of age on wearable use, independent of digital health literacy, was also significant (coefficient = -0.0928, 95% CI: -0.1331 to -0.05370, *p* <.001). The total effect of age on wearable use (coefficient = -0.1084, 95% CI: -0.1491 to -0.06960, *p* <.001) indicates that age is negatively associated with wearable use, with digital health literacy partially mediating this relationship.

## Discussion

### Principal Findings

The study investigates the associations between sociodemographic factors, digital health literacy, and the use of wearable technologies for health among adults in Germany. The major findings reveal that 24% of adult internet users in Germany report using wearables for health. Sociodemographic disparities in wearable use were identified; specifically, older adults (65+), those with lower education levels, smaller households, lower income, and those living in smaller cities or former East Germany showed a lower prevalence of wearable use. Wearable users tend to be younger and exhibit higher levels of digital health literacy, as determined by the eHEALS questionnaire, compared to non-users. The study identified a digital generational gap: among younger adults (18–40), digital health literacy was not a significant predictor of wearable use, indicating high adoption rates regardless of digital health literacy for Digital Natives. Whereas, for adults aged 41 and above, digital health literacy was a significant positive predictor for wearable use, highlighting its importance in technology adoption among older generations.

As a supplementary analysis, we conducted a mediation analysis to explore the potential role of digital health literacy as an intermediary in the relationship between age and wearable use. Results indicated that digital health literacy partially mediates this relationship, suggesting that as age increases, lower digital health literacy may contribute to lower wearable adoption. This insight supports our primary findings on sociodemographic disparities by providing an understanding of the indirect pathways through which age affects wearable use. However, as this was an exploratory analysis, future research should confirm these findings in longitudinal or intervention-based studies to establish the causative mechanisms underlying these associations.

Our analysis revealed that participants excluded due to incomplete eHEALS data were significantly older compared to those included in the final sample. However, despite this difference, the final sample still includes a substantial proportion of 65+ older adults (*n* = 297), suggesting that older adults remain well-represented in the analysis. This minimizes the risk of selection bias due to age and ensures that our findings remain relevant for understanding wearable adoption across all age groups.

### Implications & Recommendations

Wearable devices provide unique capabilities for real-time health monitoring, feedback, and self-regulation, which can support individuals in making health-related decisions (Vetrovsky et al., 2022). However, the adoption of wearables is influenced by various factors beyond their technological features, including SDoH, DDoH, and individual determinants, such as motivation, engagement, and habitual behaviors (Richardson et al., 2022). Wearables represent an innovative approach to health promotion, complementing broader methods like behavioral interventions, educational programs, and policy-based initiatives (Brickwood et al., 2019). Healthy lifestyle and educational programs often aim to change underlying health beliefs and behaviors, while policy initiatives can create environments that make healthy choices more accessible or socially encouraged. Compared to these approaches, wearables primarily rely on motivation, engagement, and habits, which may limit their impact on those who are not already inclined toward health-promoting behaviors (Wang et al., 2020). This dependency on user engagement may limit adoption among those less familiar with or inclined toward health technologies, highlighting the need to understand specific barriers to wearable use across different populations.

The findings of this study contribute to a better understanding of the sociodemographic-related contextual factors of wearable use for health promotion and disease prevention in Germany and provide insights into the broader consideration of digital health literacy and technology adoption. First and foremost, the study adds to the evidence that digital health literacy is an important element of technology adoption (van der Vaart et al., 2019). It underlines the role of digital skills in navigating and engaging with health technologies (Cameron D Norman & Harvey A Skinner, 2006). Our findings align with previous research showing that individuals with higher digital health literacy are more likely to adopt and integrate digital health interventions into their daily lives (Liu et al., 2022). These findings suggest that digital health literacy plays a critical role in facilitating the adoption of wearable technologies and other digital health interventions, emphasizing the importance of addressing literacy gaps in public health strategies.

Thus, public health interventions and policies that include technological approaches should consider leveraging strategies to enhance digital health literacy, particularly among disadvantaged demographic groups that may face barriers (Cheng et al., 2020). Educational interventions to enhance digital health literacy have shown positive results, providing individuals with the knowledge and skills necessary to interact with digital health tools and resources. These interventions may include targeted training workshops, community outreach programs, and educational campaigns to enhance digital skills and promote informed health decision-making (De Main et al., 2022).

The observed digital generational gap reinforces the notion that the “Digital Native” (born after 1980) has been immersed in technology from an early age, naturally exhibiting higher adoption rates, reflecting the greater tech-savviness of younger demographics (Rosli et al., 2016). For the “Digital Immigrant” (born before 1980), the rapid evolution of technology can be overwhelming, leading to feelings of unfamiliarity and, in some cases, resistance to embracing new digital tools (Fang et al., 2023). Although digital health literacy is an important factor in wearable adoption, it may not fully explain the non-use among older adults. In addition to digital health literacy, aesthetic preferences, comfort, and privacy concerns are frequently cited barriers to wearable use in older populations (Lee et al., 2021). Older adults may feel that wearable devices do not align with their personal style, or they may have reservations about data privacy, fearing that wearable technology could compromise their personal information (Pan et al., 2024). These preferences and concerns highlight that wearable adoption is not solely a matter of competency but also involves motivation or other individual factors. Similarly, the observed regional differences (city size & East/West Germany) could be explained by the availability of green or blue spaces, rurality, and infrastructure maturity, as well as the composition of the population (Hoffmann et al., 2022). Understanding this generational gap and regional differences is necessary for tailoring digital interventions that cater to the learning styles, preferences, and apprehensions of each community (Fang et al., 2023; Friemel, 2014) and avoid inducing “intervention-generated inequalities” (Lorenc et al., 2013). Our findings suggest efforts to reduce the digital divide should prioritize enhancing digital health literacy, particularly among older adults and those in rural or underserved regions. Designing wearables with simplicity and user-friendliness in mind could further lower barriers to entry (Jahnel et al., 2024). Tailored educational programs that address these groups’ unique challenges, such as targeted workshops or accessible community resources, could be effective in bridging technology adoption gaps (Cheng et al., 2020). Additionally, interventions should consider the specific technological and individual needs or concerns of each demographic to make wearable use more appealing and accessible (Jahnel et al., 2024).

On the other hand, wearable users may represent a subset of individuals who are already health-conscious or more engaged with digital tools. This potential self-selection bias underscores the importance of examining whether wearables reach diverse populations or remain tools predominantly used by those who are already motivated. Understanding adoption patterns among less-engaged groups could inform strategies to increase inclusivity, ensuring that wearable technologies are accessible and relevant to a broader audience.

The adoption of wearables is also shaped by the economic interests of wearable producers and commercial factors. It is also important to consider that these wearable companies often determine product design, pricing, accessibility, and affordability (Jahnel et al., 2024). Additionally, the marketing strategies of wearable companies may shape public perceptions of these devices, framing them as essential for health improvement, which could lead to overreliance on commercial products for health monitoring (Pan et al., 2024). Recognizing these factors is essential for understanding the broader impact of wearables on public health and for developing policies that promote equitable access to digital health tools.

It is essential to recognize that bridging this gap is not merely about introducing new technologies but involves providing comprehensive support systems that empower diverse sociodemographic groups to navigate and harness the benefits of wearables effectively (Friemel, 2014). Such support systems could include tailored educational programs to improve digital health literacy, such as workshops that teach individuals how to use health apps or medical apps and interpret their data (Maaß et al., 2022). Additionally, providing accessible technical support—via community health centers or virtual assistance—can address usability challenges that may discourage adoption. Community-based interventions, such as peer-led training sessions or collaborations with local organizations, could further support groups with lower technology adoption rates. Ensuring privacy protections and designing user-friendly interfaces can also help mitigate data protection concerns and improve adoption among older adults or less tech-savvy individuals. These strategies are crucial for addressing barriers to technology adoption, fostering inclusivity, and contributing to more equitable access to digital health resources. While the observed associations between sociodemographic factors, digital health literacy, and wearable use may appear modest, they offer important insights into digital health disparities. Identifying even slight variations in wearable adoption across age, education, and regional demographics can inform public health efforts to better support populations with lower technology adoption rates. These findings highlight the potential for targeted awareness and educational efforts to reduce digital health disparities and encourage health-promoting technology use in diverse sociodemographic groups. Understanding these patterns is crucial for shaping public health strategies that address barriers to technology adoption, ultimately contributing to more equitable access to digital health resources.

### Limitations

The cross-sectional nature of the study design limits our ability to establish causal relationships. While we can identify associations between variables, we cannot conclude the determinants of wearable use. Additionally, while efforts were made to align the sample’s composition with key population demographics (such as age, sex, and education parameters), it is important to acknowledge that complete representativeness may not have been achieved due to inherent limitations in sampling methods and potential biases (De Santis et al., 2024). Another consideration is the reliance on self-reported data, introducing the potential risk of recall bias and subjectivity. Digital health literacy, a key focus of this study, was self-assessed by participants. While self-assessment provides insights into individuals’ perceptions of their digital health literacy, it does not constitute a comprehensive evaluation of their actual competencies. Furthermore, it should be noted that the survey dataset had a limited number of socioeconomic and demographic variables available, potentially constraining the depth of analysis in exploring certain contextual factors. For example, the absence of ethnicity data, which may have provided valuable insights into potential disparities in wearable adoption and digital health literacy across ethnic groups. The exclusion of non-German speakers may limit the generalizability of the findings to a broader demographic. Additionally, the dichotomization of wearable use in this study was restricted to current usage and did not include past use. We acknowledge that the non-user group may include individuals who use smartphone apps for health tracking, which share some functions with wearables. While this could introduce heterogeneity in the reference group, our primary focus was on wearable adoption as a novel technology rather than on self-monitoring behavior. This distinction justified treating wearable users as a separate category which justified treating wearable users as a distinct category. Furthermore, while our stratified analysis by age provides preliminary insights, the inclusion of interaction terms could formally test for effect modification. However, due to sample size considerations, adding multiple interaction terms was not feasible, as this could compromise the stability of our estimates. Future studies with larger samples would benefit from including interaction terms to better assess whether associations vary significantly across subgroups.

Despite these limitations, our results allow us to answer our research questions with certain caveats. The robustness of the statistical analyses and the consistency of findings across multiple sociodemographic factors enhance the study’s internal validity. Additionally, the overrepresentation of older adults, while posing challenges to generalizing wearable use prevalence to the national average, contributes to the strength of the study in offering insights into a demographic group often underrepresented in similar research designs.

### Future Research Directions

Moving forward, several promising avenues for future research on the use of wearables for health can be identified. Integrating Ecological Momentary Assessment (EMA) into future studies could offer a more nuanced understanding of the day-to-day variations in wearable usage patterns (Brannon et al., 2016). EMA involves collecting real-time data on participants’ behaviors and experiences in their natural environment, providing insights into how environmental contextual factors influence wearable use patterns on a daily basis (Brannon et al., 2016). This approach could complement traditional survey methods, offering a richer understanding of the interplay between individual characteristics, environmental factors, and the use of wearable devices in real-world scenarios. Future research employing longitudinal designs would be beneficial in understanding how determinants of wearable use evolve over time.

Additionally, incorporating more objective assessments of digital health literacy, such as practical evaluations or standardized tests, would provide a nuanced understanding of participants’ actual competencies in navigating digital health technologies. One such tool is the Digital Health Literacy Instrument (DHLI), which assesses a range of digital health competencies, including operational skills, information searching, evaluating the reliability and relevance of online health information, and communicating using digital platforms (van der Vaart et al., 2017). Employing tools like the DHLI would provide a more comprehensive understanding of participants’ digital health literacy and inform strategies to address potential gaps. Further research could also explore the cultural, societal, and behavioral influences shaping technology adoption and the motivations and barriers that drive or hinder individuals’ decisions to use wearables. These present another input on individual contextual factors beyond demographic considerations. Further research should also take regional living conditions into account in order to identify potential risk groups for digital health inequalities and enable customized measures for health promotion and disease prevention.

Moreover, ensuring inclusive research practices, such as actively engaging participants who communicate in languages other than German, would enhance the external validity of findings, contributing to a more comprehensive understanding of wearable adoption across diverse demographic groups. Nevertheless, participatory research, co-creation approaches, and human-centered design are explicitly meaningful for developing appropriate, inclusive, and effective measures for health promotion in specific demographic settings.

## Conclusions

The study investigated the role of sociodemographic factors and digital health literacy in wearable use for health among adults in Germany. The findings identified significant social differences in wearable adoption and digital health literacy in the German population, particularly highlighting the digital generational gap in technology use. Data showed that digital health literacy is a significant predictor of wearable adoption among older adults (41–64 and 65+), but not among younger adults (18–40), where wearable use was high regardless of digital health literacy levels. These results highlight the importance of considering age-related differences when examining patterns of health technology adoption and understanding how digital health literacy contributes to these variations. By providing insights into these disparities, this study contributes to a more nuanced understanding of health technology adoption and its implications for equity and inclusivity.
